# Association between the Ratio of FEV_1_ to FVC and the Exposure Level to Air Pollution in Neversmoking Adult Refractory Asthmatics Using Data Clustered by Patient in the Soonchunhyang Asthma Cohort Database

**DOI:** 10.3390/ijerph15112349

**Published:** 2018-10-24

**Authors:** Sol Yu, Sujung Park, Choon-Sik Park, Sungroul Kim

**Affiliations:** 1 Department of Environmental Sciences, Soonchunhyang University, Asan 31538, Korea; solsol0914@gmail.com (S.Y.); psj57732398@gmail.com (S.P.); 2 Division of Environmental Health Research, National Institute of Environmental Research, Incheon 22689, Korea; 3 Department of Internal Medicine, Soonchunhyang Bocheon Hospital, Bucheon 22972, Korea; mdcspark@hanmail.net

**Keywords:** air pollution, refractory asthma, spirometry, FEV_1_, FVC

## Abstract

Using real-world cases, asthma-related clinical data were clustered by patient; 5% of all asthmatics were found to have refractory asthma (RA) with a relatively low lung function (forced expiratory volume in 1 s/forced vital capacity (FEV_1_/FVC) less than 80%). Using a multilevel study design for clustered spirometry data observed between 2005 and 2014, we evaluated the association between changes in the ratio of FEV_1_ to FVC and variations in acute exposure to air pollution. We analyzed 2310 episodes of RA from 214 neversmoking patients. In spring, a 1 µg/m³ increase in concentration of particles ≤10 μm (PM_10_) on Lag 1 significantly reduced the ratio by 0.4% (95% confidence interval (CI): 0.1–0.7%) after adjusting for sex, age, body mass index (BMI), and total Immunoglobulin E (IgE) level. Unit (ppb) increase in SO_2_ concentration on Lag 3 and 4 in fall and on Lag 6 in winter significantly reduced the ratio by 2 to 3% (*p* < 0.05). We found that acute exposure to PM_10_ in spring or SO_2_ in fall or winter were positively associated with lung function drop indicating necessity of control strategies of target air pollutant source by season to protect susceptible population.

## 1. Introduction

According to the American Thoracic Society (ATS) [1], refractory asthma is defined as asthma which is “difficult to treat with high doses of corticosteroids, requires high levels of medication to control persistent symptoms, and is subject to exacerbations and persistent airflow obstructions despite the use of high or maximal doses of medication”.

Acute exacerbations of refractory asthma among the elderly can require lifesaving treatments. Thus, knowing the risk factors for refractory asthma (RA) is important to minimize emergency medical interventions and to maintain normal activities of daily living among the elderly [2]. Several previous studies have demonstrated that asthma morbidity is associated with several different environmental and behavioral factors, including viral infections, sensitization to allergens, weather conditions, exercise, smoking, and diet [3,4,5,6,7,8].

Ambient air pollutants also have been considered important triggers, and this is supported by studies published over the last several decades [9,10,11]. Recently, a meta-analysis of 22 studies among 208 publications performed between 2000 to 2016 showed significant associations of major pollutants, including ozone (O_3_), nitrogen dioxide (NO_2_), carbon monoxide (CO), and particulate matters with a diameter of less than 2.5 micrometers (PM_2.5_), with exacerbations of asthma requiring emergency department visits (EDVs) and/or hospitalization [11]

According to a study conducted in the United Kingdom, a susceptible population’s daily walking on streets polluted with high PM_2.5_ and black carbon concentrations consistently reduced forced expiratory volume during the first second (FEV_1_) and forced vital capacity (FVC) [9]. This association between selected outdoor air pollutants and worsening of asthma symptoms has been observed in recent studies [10]. A recent study conducted in Los Angeles demonstrated an association between acute systemic decrease of FEV_1_ following exposure to traffic-related particulate matter [11]. This association was modified by human immunological response [12,13].

Health effects of exposure to such risk factors may be different by patient due to his/her baseline health status. Although, considering difference of symptom of asthma by patient likely provides a foundation from which to understand disease causality and to develop exposure source management approaches that lead to improved asthma control while decreasing the risk of severe asthma results (e.g., exacerbations and loss of pulmonary function) [14,15], studies examining the associations between lung function in patients with RA and ambient air pollution and meteorological conditions that take into account the clustering within each patient are limited.

In this study, we evaluated the association between variation in acute exposure levels to ambient air pollution and meteorological conditions, and changes in the FEV_1_/FVC ratio, also known as the Tiffeneau–Pinelli index, used in the diagnosis of obstructive and restrictive lung disease, using a multilevel model assuming that the observations derived from the clinical measurements were clustered by patient.

## 2. Materials and Methods 

### 2.1. Study Population

We used clinical data that was collected from adults with asthma (age, ≥20 years) who had been registered with the Genome Research Center for Allergy and Respiratory Diseases, Soonchunhyang University Bucheon Hospital, South Korea since 2005. All participants were never-smokers and had been diagnosed according to the Global Initiative for Asthma (GINA) guidelines [16] and RA, according to the American Thoracic Society (ATS) definition [17]. A spirometry was performed using a Vmax Series 2130 Autobox Spirometer (Sensor Medics, Yorba Linda, CA, USA). Baseline FVC, FEV_1_, forced expiratory flow rate between 25% and 75% FVC (FEF 25–75%) and carbon monoxide diffusing capacity were obtained when a bronchodilator was not used within the previous 8 h. Postbronchodilator FEV_1_, and FVC were measured 20 min after inhalation of a short-acting bronchodilator. 

**Figure 1:**
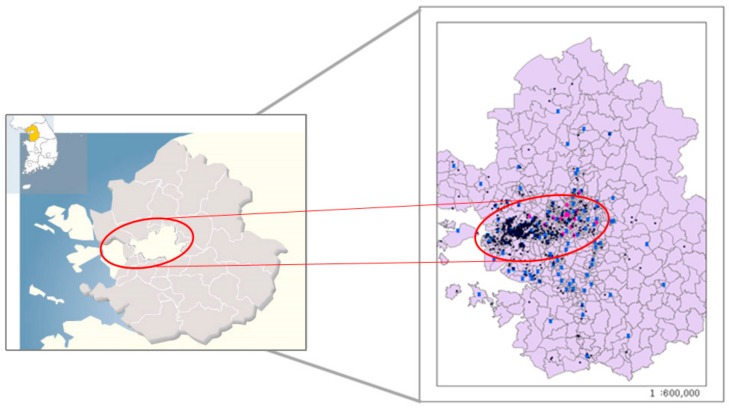
Locations of study areas (Seoul: white area in center, Incheon: white area in left side and Bucheon: between Incheon and Seoul) (**Left**) and residential (black dots) or air monitoring (blue dots) sites obtained from geographical information system (**Right**).

We included only those participants with an abnormal FEV_1_/FVC ratio (Tiffeneau–Pinelli index) less than 80% [18]. Participants were resident in Seoul (population, 10.3 million), the largest metropolis and capital of South Korea, Incheon (population, 3 million), one of metropolitan cities in South Korea, bordering Seoul and Gyeonggi province, or Bucheon (population, 0.8 million), a satellite city of Seoul in Gyeonggi province (Figure 1). Participants who had experienced multiple episodes of asthma exacerbation, requiring an increase in medication, including systemic steroids, were recruited from Soonchunhyang Hospital between 2005 and 2014. with multiple episodes of exacerbations requiring an increase in asthma medication, including systemic steroids. Study participants conducted a spirometry test at their visit according to an international guideline [19]. Our final data had 2310 episodes from the 214 neversmoking RA patients. 

We excluded patients who had chronic obstructive pulmonary disease (COPD), vocal cord dysfunction, obstructive sleep apnea, Churg–Strauss syndrome, cardiac dysfunction, or allergic bronchopulmonary aspergillosis (ABPA). Demographic information was collected from registration documents submitted by the patients on their first hospital visit during the study period. Participants were followed for a minimum of 2 years. Original study procedures relating to human participants and informed consent were approved by the research ethics committee of Soonchunhyang University Bucheon Hospital (SCHBC_2014_07_028).

### 2.2. Meteorological and Air Pollution Data

Meteorological data, including mean daily temperature, relative humidity, and air pressure, were obtained at national monitoring sites closest to the hospital [20]. Air pollution measurements were obtained from the national ambient monitoring station located closest to homes of the patients, as previously described [20]. Daily (24-h) averages for PM_10_, sulfur dioxide (SO_2_), and nitrogen dioxide (NO_2_) levels were used, whereas 8-h running averages were used for ozone (O_3_) and carbon monoxide (CO) levels. We had approximately 45 national air quality monitoring sites at districts or counties of each city (*n* = 4, 15, and 26 at Bucheon, Incheon and Seoul at the time of our study, i.e., 30 December 2015). The median (5~95 percentile) distance from monitoring stations to patient’s address was 1.63 (0.72~2.99) km.

### 2.3. Data Analysis

Descriptive statistics (frequencies, percentages, means, medians, and interquartile ranges) and plots were used to describe the study population. We determined differences in demographic characteristics and FEV_1_/FVC ratios according to normal body mass index (BMI) or interquartile range of total immunoglobulin E (IgE) using Fisher’s exact test and the Mann–Whitney *U* test or Kruskal–Wallis test. Statistical significance was based on a *p*-value less than 0.05. 

To evaluate the impact of exposure to ambient air pollution and meteorological conditions on exacerbation of RA, we used a multilevel model procedure to account for the clustered data (either air pollution exposure or health outcome) within one patient. A random intercept model was used and the coefficients and standard errors were estimated under restricted maximum likelihood estimation (REML) with unstructured autocorrelation. We assumed that there was a two-level structure within the data. 

The analyses included episode level (level-1) and individual level (level-2). The episode level included each spirometer results with exposure level to air pollution matched time in each patient, centered by the seasonal median concentrations. The individual level was defined as per personal total IgE levels, sex, BMI, and age at first pulmonary function test (PFT_age). Using the combined level-1 and level-2 data sets, multilevel modeling provides a way to analyze the degree to which the effects of episode level variables vary systematically or randomly as a function of the individual (level-2) variables [21]. 

We assumed that the intercepts for different patients were normally distributed. To estimate the dependency of the measurements of lung function of each patient, we calculated the intraclass correlation coefficient (ICC), defined as the variance between patients divided by the total variance (the summation of the variance between patient and the variance within patient). The lower the variance within the groups, the higher the ICC [21]. 

Each episode corresponded to a diagnosis of exacerbation with shortness of breath, wheezing, or chest tightness, and decrease in expiratory airflow; clinically, each episode was defined as post-bronchodilator peak expiratory flow (PEF) or FEV_1_ of <80% of the predicted value or personal best and usage of high doses of inhaled steroids. We constrained an “episode” to have a frequency no greater than one visit per month to avoid attributing follow-up visits to new independent episodes [22]. The seasonal distributions of meteorological conditions and air pollution concentrations on the day of admission of each episode were compared using the Wilcoxon rank-sum test. 

The effects of meteorological factors and air pollution levels on asthma exacerbation were evaluated on the day of admission (Lag 0) and up to 6 days before admission (Lag 1 through Lag 6), using a multilevel model. Log-transformation was conducted for the dependent variable, FEV_1_/FVC, in the regression analysis to account for the right-skewed distribution. The geometric mean ratio of FEV_1_/FVC was calculated to estimate the increased risk of exacerbation of poor lung. We identified 214 patients with an abnormal FEV_1_/FVC ratio (below 80%) function according to unit increases in air pollution levels and changes in meteorological conditions. 

Our first regression model assessed the lung function of never-smoking patients, expressed by FEV_1_/FVC ratio, relative to air pollution and meteorological data, after controlling for seasonality. We stratified the data into four seasons: spring (March–May), summer (June–August), fall (September–November), and winter (December–February) with respect to the monthly median temperature. Then, as a final model, we included the same environmental risk factors as stated above and analyzed the effect of exposure to air pollution and meteorological conditions on changes in FEV_1_/FVC. We adjusted for overall quantity of IgE in the blood (total IgE), sex, age, BMI, and seasonality.

Multiple sensitivity analyses were conducted. First, in addition to the model described above, we ran two separate models for those never-smoking RA patients with a FEV_1_/FVC ratio below 70% and for those whose ratio ranged from 70 to 80% separately from our overall never-smoking RA patients’ data. Second, using former-smoking RA patient data, we also examined the impact of exposure to the air pollution and meteorological factors on lung function as we mentioned above. All analyses were conducted using the SAS 9.3 package (SAS Institute, Cary, NC, USA).

## 3. Results

**Figure 2:**
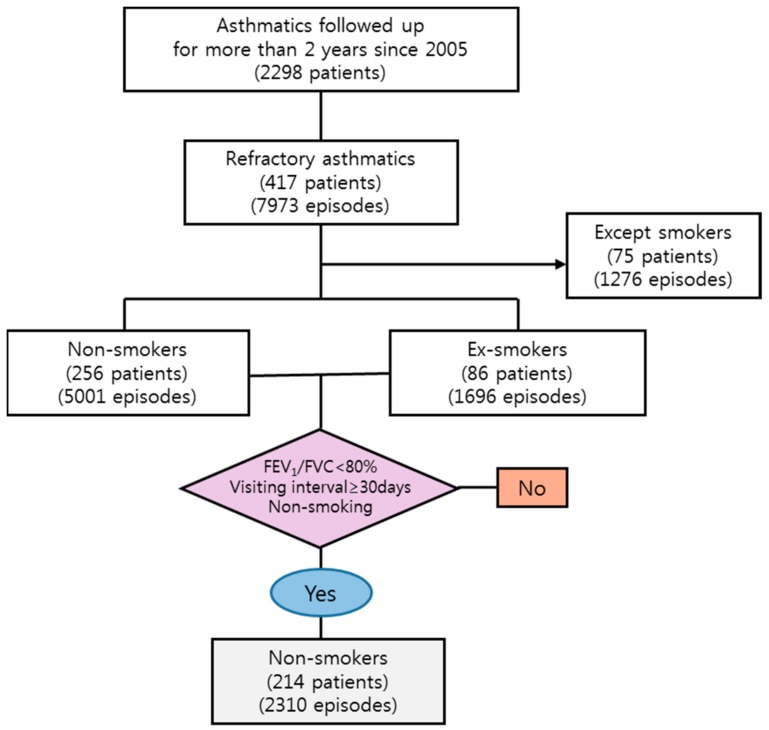
Flow chart for selecting process of study population from the cohort database of Department of Internal Medicine, Soonchunhyang Bucheon hospital. FEV_1_: forced expiratory volume during the first second; FVC: forced vital capacity.

We identified 214 patients with an abnormal FEV_1_/FVC ratio (below 80%), who lived within the study area, and presented to the study hospital within the study period with multiple episodes of exacerbations requiring an increase in asthma medications including systemic steroids. Our final data comprised 2310 episodes relating to these 214 never-smoking patients (Figure 2).

Among never-smoking RA patients, women were predominant (86.5%); their median (interquartile range, IQR) age at the time of spirometry was 65.2 years (56.4–72.4 years) and they were approximately 7 years older than the men (58.0 years (47.4–70.4 years), (*p* < 0.001)). Median (IQR) BMI was 24.7 (23.6–27.6) kg/m^2^ for male patients and 24.4 (22.4–26.4) kg/m^2^ for female patients. The median (IQR) of the FEV_1_/FVC ratio was lower among men than women (68.0% (60.0–73.0%)) and 72.0% (66.0–76.0%)) (*p* < 0.05) (Table 1). 

In addition, among never-smoking RA patients, we observed that the median ratio was relatively smaller in patients with lower BMI (<25 kg/m^2^; 71.0% [63.0–75.0%]) compared to patients with higher BMI (72.0% [67.0–76.0%]). The median ratio was also lower (69.0% [62.0–75.0%]) in those whose total IgE level was in the lowest quartile (<30 IU/mL) than in the other quartiles (≤30~<130 IU/mL, 130~< 260 IU/mL, or ≥260 IU/mL) (Table 2). 

Table 3 shows descriptive statistics relating to the levels of air pollution and meteorological conditions at the time of admission (Lag 0) of neversmokers. Distributions of the levels of these environmental factors differed according to season (*p* < 0.05). 

**Figure 3:**
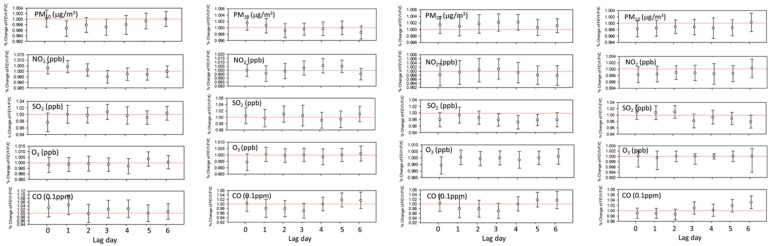
Association of FEV_1_/FVC variation with exposure level to air pollution in neversmoking patients—spring, summer, fall, winter. Results were adjusted for meteorological condition, sex, age at the first pulmonary function test (PFT) and Total_IgE level.

The ICC was 0.59 and we constructed modeling taking into account the correlation between lung function measurements of each patient over time. Among neversmokers, in spring, after adjusting for sex, age, BMI, and total IgE level, a 1 µg/m³ increase in PM_10_ concentration on Lag 1 significantly reduced the FEV_1_/FVC ratio by 0.4% (95% confidence interval (CI): 0.1–0.7) (*p* < 0.05). A 1 ppb increase in SO_2_ concentration on Lag 3 or Lag 4 in fall and on Lag 6 in winter significantly reduced the ratio by 2 to 3% (*p* < 0.05), after controlling for other explanatory variables. In winter, a 1 °C decrease in temperature on Lag 4 significantly reduced the ratio by 0.3% (Figure 3). In our model, total IgE level had a negative but non-significant effect on the change in the ratio (data not shown). 

## 4. Discussion

In this study, we evaluated the association between level of exposure to atmospheric environmental risk factors and deterioration in pulmonary function among never-smokers using mixed models, after controlling for seasonality, first PFT age, sex, and total IgE level. Our results demonstrated that, especially SO_2_ exposure in autumn and winter and PM_10_ in spring was the seasonal potential risky air pollutant associated with reducing lung function among refractory asthmatics. 

Our research outcomes were similar to those of previous studies. According to an experimental study [23] conducted with 38 participants in Seattle, mean (standard deviation) negative change (% before–after) in FEV_1_ was 17.2% (10.9%) among SO_2_ responders (*n* = 22) and 1.9% (3.5%) among SO_2_ non-responders (*n* = 16). Moreover, corresponding plasma-carotene concentrations were inversely associated with PEF values, and ascorbate concentrations were inversely associated with FEV_1_ and FEV_1_/FVC. Participants in this study were exposed to an SO_2_ level of 500 ppb for 10 min on a treadmill moving at 2 miles per hour up a 10% gradient, conditions assumed to correspond with 8 h’ exposure to 10 ppb of SO_2_ in air. Since our seasonal ambient SO_2_ concentrations ranged from 5 to 8 ppb, we concluded that our observational study results were supported by these experimental study results. 

Our study showed that a PM_10_ exposure in spring was associated with a decrease in FEV_1_/FVC ratio. Similar results (FVC drop rather than FEV_1_/FVC) from multilevel-based mixed models were previously reported in Europe [24,25]. Due to absence of our nationally available PM_2.5_ monitoring data corresponding to our study period, it is difficult to directly compare our results with the recent European results. It will be possible for us to obtain more meaningful information when we undertake further modeling to include spatiotemporal distributions of mobile pollutant-related indicator materials, such as PM_2.5_ or black carbon concentrations.

Since a relatively large number of studies have been conducted with child rather than adult asthma patients, although direct comparisons are difficult, we compared our study results with those from a child study. A recent study of children living in Hawaii demonstrated that in the group exposed to high concentrations of SO_2_ (1.2 ppb) and PM_2.5_ (7.2 μg/m^3^), the proportion of those whose FEV_1_/FVC ratio was less than 80% was 1.3 times higher than that of the controls [26]. In our study, we did not find a statistically significant relationship between total IgE level and decrease in lung function. Further replication is required as few studies have evaluated this relationship. 

Our study has several strengths. We used a clustered measures design, which is a repeated measure design, to assess asthmatics that were vulnerable to current levels of air pollution in South Korea. This design reduced between-subjects variability and increased the statistical power. This suggested that multilevel analysis using mixed models that consider the high correlation of each within-subjects lung function data is useful. In addition, spirometry testing was consistent, which excludes between-instrument variability in the lung function test 

However, the results of our study should be interpreted with care due to the following limitations. First, the hospital data used in this study did not include information on socioeconomic status or level of exposure to secondhand smoke. This information was recorded in written medical files, which could not be digitized for this study. In the future, we intend to analyze the association between changes in pulmonary function and level of exposure to air pollution more precisely, by including these additional variables. In the future, we intend to analyze the association between changes in pulmonary function and level of exposure to air pollution more precisely, by including these additional variables. Second, although this study was limited to data obtained from three hospitals, our study area included two of the largest cities in South Korea, Seoul, and Incheon, and their satellite city, Bucheon. Therefore, our results may not be generalized to adult asthma sufferers living in other urban metropolitan areas of South Korea. Third, we included only outdoor exposure data in our study, and it is likely that the participants spend considerable amounts of time indoors. In future research, both indoor and outdoor air quality and participants’ behavior patterns should be taken into consideration to obtain a more precise analysis.

Nevertheless, in this study, our clinically clustered data by patient was characterized by quantitative changes in pulmonary function obtained from lung function tests at each hospital visit. These data were analyzed to identify air pollutants affecting changes in lung function according to the season, and, based on this information, basic information on the management of air pollutant sources for sensitive groups was presented. In addition, this study quantitatively suggests that multilevel analysis using mixed model taking into account high correlation of each individual’s lung function results is useful. 

## 5. Conclusions

We found that acute exposure to PM_10_ in spring or SO_2_ in fall or winter was positively associated with lung function drop among RA with 1- or 3-day lags. Our results provided quantitative evidence of necessity of control strategies of target air pollutant source for sensitive group by season. 

## Figures and Tables

**Table 1:** Distributions of age, lung function, or total immunoglobulin E (IgE) level according to sex in neversmoking asthmatics.

	Overall (*n* = 2310) Median (IQR)		Male (*n* = 312) Median (IQR)		Female (*n* = 1998) Median (IQR)		*p*-Value *
Episode Level (Level one, *n* = 2310)							
FEV_1_ (Liter)	1.6	(1.3~2.0)	2.2	(1.6~2.6)	1.6	(1.3~1.9)	*p* < 0.001
FEV_1_ (%)	81.0	(69.0~90.0)	70.5	(57.0~82.5)	82.0	(71.0~91.0)	*p* < 0.001
FVC (Liter)	2.3	(2.0~2.4)	3.2	(2.7~3.7)	2.2	(1.9~2.6)	*p* < 0.001
FVC (%)	83.5	(74~92)	79.0	(69.5~89.0)	84.0	(75.0~93.0)	*p* < 0.001
FEV_1_/FVC (%)	71.0	(65.0~76.0)	68.0	(60.0~73.0)	72.0	(66.0~76.0)	*p* < 0.001
FEF 25~75 (%)	47.0	(34.0~61.0)	45.0	(30.0~57.0)	48.0	(34.0~61.0)	*p* = 0.0044
Individual Level at baseline (Level two, *n* = 214)							
Age (years)	60.0	(51.4~67.3)	53.1	(44.0~65.7)	60.4	(52.1~67.5)	*p* < 0.001
BMI (kg/m^2^)	24.4	(22.5~26.5)	24.7	(23.6~26.7)	24.4	(22.4~26.4)	*p* = 0.01
Total_IgE (IU/mL)	127.0	(36.2~286.0)	81.7	(39.9~231.0)	131.0	(35.0~286.0)	*p* = 0.0002

* *p*-value from Mann–Whitney test for evaluation of distributions between men and women. BMI: body mass index; FEF: forced expiratory flow; IQR: interquartile range.

**Table 2:** Distributions (median (IQR)) of FEV_1_/FVC (%) according to sex, age, BMI, or total IgE level in neversmoking asthmatics (*n* = 2310 episodes).

	Sex *		Age (years) at Pulmonary Function Test *		BMI (kg/m^2^) *		Total IgE (IU/mL) **			
	Male (*n* = 312)	Female (*n* = 1998)	<60 (*n* = 1153)	≥60 (*n* = 1157)	<25 (*n* = 1380)	≥25 (*n* = 930)	<30 (*n* = 498)	≤30~<130 (*n* = 717)	≤130~<260 (*n* = 459)	≥260 (*n* = 636)
FEV_1_/FVC (%) Median (IQR)	72.0 (66.0~76.0)	68.0 (60.0~73.0)	72.0 (66.0~76.0)	70.0 (63.0~75.0)	71.0 (63.0~75.0)	72.0 (67.0~76.0)	69.0 (62.0~75.0)	72.0 (65.0~76.0)	72.0 (66.0~76.0)	72.0 (66.0~76.0)
*p*-value										

* *p*-value from Mann–Whitney test, ** *p*-value from Kruskal–Wallis test. BMI: body mass index; IgE: immunoglobulin E.

**Table 3:** Descriptive summary of baseline meteorological factors and air pollutants in neversmoking asthmatics.

	Overall	Spring (March, April, May)	Summer (June, July, August)	Fall (September, October, November)	Winter (December, January, February)	*p*-Value *
	Median (IQR)	Median (IQR)	Median (IQR)	Median (IQR)	Median (IQR)	
**Meteorological factor**						
Temperature (°C)	14.0 (4.2~21.4)	11.1 (6.7~15.6)	23.2 (21.4~25.2)	16.2 (11.9~20.5)	0.2 (−3.7~3.9)	*p* < 0.001
Relative humidity (%))	66.4 (54.3~77.1)	61.2 (50.7~70.7)	80.0 (70.2~86.7)	65.7 (55.8~73.3)	58.2 (46.8~70.4)	*p* < 0.001
**Air pollutant**						
SO_2_ (ppb)	5.8 (4.2~8.0)	6.4 (4.7~8.3)	4.5 (3.2~5.9)	5.3 (4.1~6.8)	7.9 (6.1~10.8)	*p* < 0.001
PM_10_ (μg/m^3^)	53.3 (36.0~75.8)	62.3 (45.5~83.7)	44.2 (27.0~61.1)	48.7 (32.3~64.8)	59.5 (45.5~84.5)	*p* < 0.001
NO_2_ (ppb)	36.4 (26.0~46.8)	39.2 (28.6~48.6)	30.1 (22.5~39.4)	36.3 (27.0~46.1)	41.0 (29.0~53.7)	*p* < 0.001
CO (0.1 ppm)	5.6 (4.1~7.8)	5.6 (4.4~7.2)	4.3 (3.6~5.7)	5.4 (4.0~7.6)	7.8 (5.7~11.1)	*p* < 0.001
O_3_ (ppb)	18.0 (10.7~26.7)	25.0 (18.0~31.9)	22.8 (14.7~31.9)	16.2 (11.9~20.5)	10.2 (6.3~15.2)	*p* < 0.001

* *p*-value from Kruskal–Wallis test for evaluation difference of distributions among four seasons. PM_10_: particulate matters with a diameter of less than 10 micrometers
